# Independent Association of Physical Activity with Nonalcoholic Fatty Liver Disease and Alanine Aminotransferase Levels

**DOI:** 10.3390/jcm8071013

**Published:** 2019-07-10

**Authors:** Dong Kee Jang, Jung Soo Lee, Jun Kyu Lee, Yeo Hyung Kim

**Affiliations:** 1 Department of Internal Medicine, Dongguk University College of Medicine, Dongguk University Ilsan Hospital, Goyang 10326, Korea; 2 Department of Rehabilitation Medicine, College of Medicine, The Catholic University of Korea, Seoul 06591, Korea

**Keywords:** non-alcoholic fatty liver disease, nonalcoholic steatohepatitis, transaminases, physical activity, exercise

## Abstract

The aim of the current study was to examine the independent association of physical activity with nonalcoholic fatty liver disease (NAFLD) and aminotransferases while adjusting for obesity and diet. Cross-sectional data from 32,391 participants aged ≥20 years in the Korea National Health and Nutrition Examination Surveys (KNHANES) was analyzed by logistic regression models and general linear models. Physical activity was assessed from the questionnaire by health-enhancing physical activity (HEPA). The physical activity was negatively associated with NAFLD and lean NAFLD after adjustment for multiple factors with an odds ratio of 0.7 (95% CI, 0.6–0.8) and 0.5 (95% CI, 0.4–0.7) comparing the most active (HEPA active) and the least active (inactive) participants. Among the participants with NAFLD, physical activity also showed an independent negative association with alanine aminotransferase (ALT) levels but not with aspartate aminotransferase levels. These independent associations were not observed when comparing the minimally active and inactive participants except for the risk of lean NAFLD. Physical activity is independently associated with the degree of hepatocellular injury in patients with NAFLD as well as the risk of NAFLD and lean NAFLD in the general population. Sufficiently active physical activity greater than a minimally active level may be needed to lower the risk of NAFLD and ALT levels.

## 1. Introduction

Nonalcoholic fatty liver disease (NAFLD) is defined as the presence of hepatic steatosis with no secondary causes of hepatic fat accumulation, such as significant alcohol consumption [1]. NAFLD has become a leading cause of chronic liver disease in Western countries [2,3]. A similar high prevalence of NAFLD has been reported in Asia, including South Korea, with a global prevalence of 25.2% [2,4]. NAFLD may progress to cirrhosis and is known to be an important cause of cryptogenic cirrhosis, and even hepatocellular carcinoma [3]. Furthermore, lean individuals with NAFLD (lean NAFLD), who are characterized by a severe histological phenotype similar to obese subjects, have received attention recently [5]. Lean NAFLD was known to be associated with an increased risk of all-cause mortality as well as metabolic syndrome [6]. Therefore, the economic and clinical burden of NAFLD is growing rapidly worldwide.

Despite the high prevalence of NAFLD, there is no well-established treatment except for weight loss [1,7]. The weight loss required for the improvement of steatosis is known to be 3–10% of body weight, which can be achieved either by hypocaloric diet alone or in conjunction with increased physical activity [1]. There is increasing evidence that individuals with lower physical activity are at higher risk of NAFLD [8,9,10,11,12,13]. However, controversy remains regarding the independent association between physical activity and NAFLD regardless of other factors, such as obesity and diet [9,11,12,13]. Furthermore, many previous studies had a small number of participants or did not adjust for confounders such as diet and central obesity.

To date, large-scale studies to clarify that physical activity is independently associated with NAFLD or lean NAFLD are limited. Additionally, to the best of our knowledge, no previous study has examined the independent association of physical activity with the level of aminotransferases, which can be an indicator of liver damage. Accordingly, the aim of the present study was to evaluate the independent association of physical activity with NAFLD and lean NAFLD in the Korean general population, adjusting for lifestyle confounders and comorbidities. We also aimed to investigate the association of physical activity with the level of aminotransferases in participants with NAFLD.

## 2. Materials and Methods

### 2.1. Study Participants

The present study used the fourth, fifth, and sixth Korea National Health and Nutrition Examination Surveys (KNHANES) database. The KNHANES Data were obtained by household interviews and standardized physical examinations administered at mobile examination centers. A nationally representative sample of the noninstitutionalized Korean general population was collected by applying a stratified, multistage, clustered probability sampling method. The KNHANES was conducted by the Korean Centers for Disease Control and Prevention and the database is available in Korean [14].

**Figure 1:**
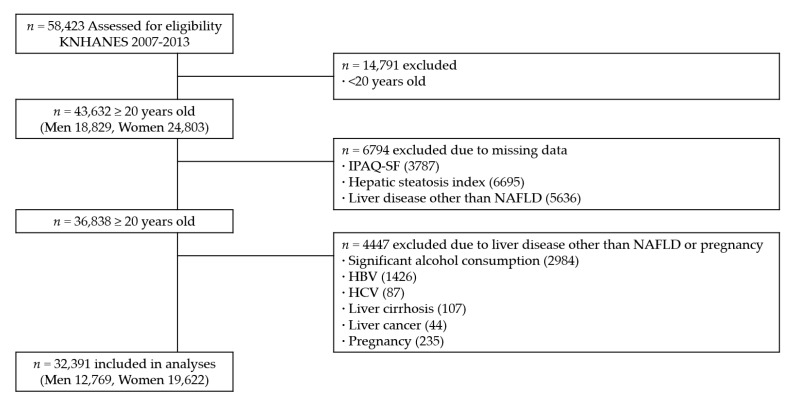
Flow diagram of participant inclusion and exclusion in the current study using data from the Korea National Health and Nutrition Examination Surveys (KNHANES IV, V, and VI). IPAQ-SF, International Physical Activity Questionnaire Short Form; NAFLD, nonalcoholic fatty liver disease; HBV, hepatitis B virus; HCV, hepatitis C virus.

A total of 58,423 participants were recruited in KNHANES from 2007 to 2013. Among the 58,423 participants, the study population consisted of 36,838 participants aged ≥ 20 years who completed the comprehensive health surveys and examinations (Figure 1). We excluded 4447 participants for the following reasons: (1) significant alcohol consumption of ≥30 g/day for men and ≥20 g/day for women (*n* = 2984) [1]; (2) positive serologic markers for hepatitis B (*n* = 1426); (3) diagnosed with hepatitis C by a doctor and/or use of medication for hepatitis C (*n* = 87); (4) diagnosed with liver cirrhosis by a doctor and/or use of medication for liver cirrhosis (*n* = 107); (5) diagnosed with liver cancer by a doctor and/or use of medication for liver cancer (*n* = 44); and (6) self-reported current pregnant state (*n* = 235). Since some individuals met more than one exclusion criterion, the final number of participants for this study was 32,391 (12,769 men and 19,622 women). The Institutional Review Board at the Korea Centers for Disease Control and Prevention approved the protocol. The ethical approval of our hospital was waived for the current study using publicly available data.

### 2.2. Definition of NAFLD

Due to the lack of imaging studies for liver in KNHANES, NAFLD was defined using a previously validated fatty liver prediction model: hepatic steatosis index (HSI) [15,16]. HSI was calculated according to the following formula: HSI = 8 × alanine aminotransferase (ALT)/aspartate aminotransferase (AST) + BMI (+2, if diabetes mellitus; +2, if female). An HSI >36.0 was used as a surrogate of NAFLD. With a cutoff point >36.0, the HSI could detect NAFLD with a specificity of 93.1% and a positive likelihood ratio of 6.5 [15].

### 2.3. Physical Activity Assessment

Health-related physical activity was evaluated by a validated Korean version of the International Physical Activity Questionnaire Short Form (IPAQ-SF) [17,18]. IPAQ-SF assesses physical activity during the last 7 days by measuring the frequency and duration of walking and moderate to vigorous activity across all contexts (i.e., work, transport, household, and leisure). Physical activity was assessed by categories for health (health-enhancing physical activity (HEPA)) into three levels: HEPA active (reaching recommendations for HEPA, sufficiently active), minimally active, and inactive [18]. The HEPA active category meets either of two criteria: (1) vigorous activity at least 3 days/week and accumulating at least 1500 metabolic equivalents (METs)-minutes per week (MET-min/week); or (2) 7 or more days/week of any combination of walking, moderate, or vigorous activities accumulating at least 3000 MET-min/week. The minimally active category meets either of the following 3 criteria: (1) 3 or more days/week of vigorous activity of at least 20 min/day; (2) 5 or more days/week of moderate-intensity activity and/or of walking of at least 30 min/day; or (3) 5 or more days/week of any combination of walking, moderate, or vigorous activities achieving a minimum of 600 MET-min/week. The inactive category meets neither the HEPA active nor minimally active criteria. Continuous scores (MET-min/week) were calculated according to the published formula [18].

### 2.4. Clinical and Biochemical Measurements

BMI (kg/m^2^) was calculated as the individual’s weight divided by the square of the height. Lean was defined as individuals with a BMI ≤25 kg/m^2^ [5,19]. Central obesity was defined by waist circumference using a cutoff point of 90 cm for men and 85 cm for women, which was previously determined in Koreans [20]. The mean daily alcohol intake was calculated from the questionnaires that recorded the frequency of drinking days and the number of drinks consumed per drinking day. The amount of alcohol consumption was also scored by the Alcohol Use Disorders Identification Test in Korea. Daily total calorie intake (kcal/day) was obtained using the 24 h recall method by trained dieticians. Sleep duration (hours/day), smoking habits (current, past, or never), and education level (≤6, 7–9, 10–12, >12 years) were recorded.

Venous blood samples were obtained after the participants had fasted for at least 8 h. Serum samples were immediately processed and transported to a certified laboratory, where they were analyzed within 24 h. An ADIVIA 1650 analyzer (Siemens, Washington, DC, USA) of Seoul Medical Science Institute (Seoul, Korea) in 2007 and Hitachi automatic analyzer 7600 (Hitachi, Tokyo, Japan) of Neodin Medical Institute (Seoul, Korea) in 2008–2013 were used for measurement of ALT and AST. Both analyzers employed the α-ketoglutarate reaction. The upper limit of normal ALT level was defined as 34 U/L in men and 24 U/L in women, and the upper limit of normal AST was defined as 32 U/L in men and 26 U/L in women, as previously determined from 411,240 Korean blood donors [21].

### 2.5. Statistical Analysis

The characteristics of the participants according to the status of physical activity and NAFLD were compared using analysis of variance (ANOVA) or Student’s *t*-test for continuous variables and the Chi-square test for categorical variables. The association between the physical activity category and NAFLD or lean NAFLD was analyzed by multivariable logistic regression models. To further investigate the relationship between physical activity and liver function, the association between the level of physical activity and aminotransferases (ALT and AST) was assessed among the participants with NAFLD. Since categorization of a variable can lead to the loss of information, we used the aminotransferase level as both continuous and dichotomous variables. The adjusted mean aminotransferases levels according to the physical activity level were obtained using general linear models. Multivariable logistic regression analyses were used to determine the independent association between physical activity category and abnormal elevation of aminotransferase levels by using previously defined cut-off values [21]. During analyses, five models were fitted to gradually reduce confounding associations. The baseline model (model 1) was adjusted for age and sex. BMI was added sequentially after age and sex in model 2. Lifestyle variables (total calorie intake, sleep duration, smoking, alcohol, education level) were added to model 3, and comorbidities (diabetes mellitus (DM), cardiovascular disease, hypertension, and arthritis) were further added to model 4, and finally central obesity was added to model 5, according to their established associations with physical activity and NAFLD and clinical relevance [3,9,10,12,16,22,23,24,25,26]. All analyses were performed by the complex sample procedures of SPSS (version 24; IBM/SPSS Inc., Armonk, NY, USA) to account for the complex sampling design.

## 3. Results

### 3.1. Characteristics of Participants

The baseline characteristics of the study participants according to the physical activity level and the presence of NAFLD are presented in Table 1 and Table 2, respectively. Of the study participants, the unweighted prevalence of NAFLD assessed by HSI was 21.5% (6968/32,391). The weighted prevalence of NAFLD was 22.3% (SE, 0.3%) in the Korean community-dwelling population aged ≥20 years. The inactive participants were more likely to be older and female as well as to have a lower BMI and lower daily calorie intake than the HEPA active participants. Moreover, inactive individuals showed an increased proportion of NAFLD, lean NAFLD, central obesity, and other comorbidities compared with HEPA active participants. On the other hand, participants with NAFLD were more likely to be older and male as well as to have a higher BMI, central obesity, and higher daily calorie intake than the non-NAFLD participants. In addition, participants with NAFLD were more likely to have comorbidities than non-NAFLD participants. In particular, DM showed a strong association with NAFLD with an unadjusted OR of 4.7 (95% CI, 4.3–5.2; *p* < 0.001).

### 3.2. Association between Physical Activity and NAFLD

Table 3 shows the negative association between physical activity level and the presence of NAFLD or lean NAFLD (for the ORs of adjusted variables other than physical activity see Supplementary Tables S1 and S2). A higher physical activity level (HEPA active) was independently associated with a lower risk of NAFLD and lean NAFLD than inactive physical activity. The risk of NAFLD was significantly lower in minimally active subjects compared with that in inactive subjects in the age- and sex-adjusted model (adjusted OR, 0.9; 95% CI, 0.8–0.97; in model 1). However, further adjustment for BMI made this association nonsignificant (adjusted OR, 0.9; 95% CI, 0.8–1.03; in model 2). The risk of NAFLD in minimally active participants compared with that in inactive participants remained nonsignificant in further adjusted models (models 3–5). The risk of lean NAFLD was significantly lower in minimally active lean individuals than in inactive lean individuals (adjusted OR, 0.8; 95% CI, 0.6–0.98; in model 5).

### 3.3. Association between Physical Activity and Aminotransferases in NAFLD Participants

**Figure 2:**
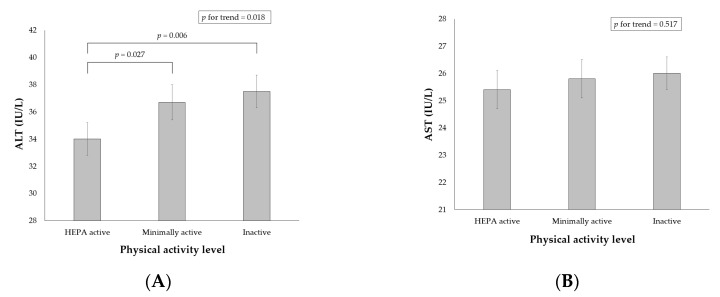
Adjusted weighted means of ALT (**A**) and AST (**B**) by the physical activity level among participants with NAFLD (*n* = 6968). Values are adjusted for age, sex, body mass index, central obesity, total calorie intake, sleep duration, smoking, alcohol, education level, diabetes mellitus, cardiovascular disease, hypertension, and arthritis. *p* values by complex-samples general linear models. ALT, alanine aminotransferase; HEPA, health-enhancing physical activity; AST, aspartate aminotransferase.

As shown in (Figure 2A), the weighted multivariable-adjusted mean ALT levels were significantly lower in HEPA active participants with NAFLD than in minimally active and inactive participants with NAFLD (34.0 ± 1.2 in HEPA active; 36.7 ± 1.3 in minimally active; 37.5 ± 1.2 in inactive participants; *p* = 0.018). However, the multivariable-adjusted mean AST levels were not different according to the physical activity level ((Figure 2B); 25.4 ± 0.7 in HEPA active; 25.8 ± 0.7 in minimally active; 26.0 ± 0.6 in inactive participants; *p* = 0.517). The associations between physical activity level and abnormal elevations in ALT and AST are presented in Table 4 (for the ORs of adjusted variables other than physical activity see Supplementary Tables S3 and S4). Physical activity level was independently associated with an abnormal elevation in ALT but not with that in AST. HEPA active participants showed a significantly lower risk for abnormal ALT levels than inactive participants across all 5 models.

## 4. Discussion

This study aimed to investigate the independent association of health-related physical activity with NAFLD and aminotransferase levels using a nationally representative database. The present study demonstrated that the sufficiently active participants who reached the HEPA active category had a lower risk of NAFLD and lean NAFLD than that of the most inactive participants after adjustment for multiple important confounders, such as obesity and diet. The intermediate level of physical activity was not associated with a lower risk of NAFLD but was associated with that of lean NAFLD. Furthermore, physical activity level showed an independent negative association with ALT levels but not with AST levels among participants with NAFLD. The HEPA active physical activity was independently associated with 3.5 IU/L lower mean ALT levels and 0.8-fold lower OR of abnormal ALT (defined as >34 IU/L in men or >24 IU/L in women) compared with inactive physical activity.

Our results indicate that a low physical activity level was independently associated with the presence of NAFLD. Several previous cross-sectional studies have reported the independent inverse association between physical activity and NAFLD [9,11]. Nevertheless, studies suggesting that obesity may be a mediator of the physical activity–NAFLD association have also been published [12,13]. The findings from a recent cross-sectional study in a Korean middle-aged population showed similar results in that decreased physical activity levels were positively associated with the prevalence of NAFLD, with prevalence ratios of 0.8 (95% CI, 0.8–0.8) for NAFLD comparing HEPA active participants to inactive participants [10]. However, the effect estimates of the highest physical activity (HEPA active) on NAFLD were higher in our study (OR, 0.7; 95% CI, 0.6–0.8) than in the previous study. The difference in effect size may be due to the differences in the study population. While the previous study included middle-aged participants who visited the healthcare center, our study included community-dwelling individuals of all age groups over 19 years.

A dose–response relationship does not seem to be established in the association of physical activity and NAFLD due to the mediating effect of obesity. In the current study, only the most active individuals (HEPA active) had a significant reduction in the risk of NAFLD, and those who were minimally active did not benefit from this risk reduction. The minimally active participants were found to have a lower risk of NAFLD than the inactive participants in the age- and sex-adjusted model. However, further adjustment for BMI abrogated this association, which suggests that a low risk of NAFLD associated with minimal physical activity was mediated by its effect on BMI, such as weight reduction by physical activities. Furthermore, the risk of lean NAFLD was significantly lower in HEPA active and even minimally active individuals than in inactive lean individuals, with a lower OR than that in all study participants. Therefore, these finding of our study are partially consistent with the previous study’s finding that obesity may be a mediator of physical activity and the NAFLD association [12,13].

The biological basis of the negative association between a high level of physical activity and a low risk of NAFLD is not well understood. One classic explanation is that physical activity can affect NAFLD by improving obesity or reducing body weight [27]. However, physical activity may have a direct effect on improving hepatic lipid metabolism and insulin sensitivity, independent of weight loss [28]. A possible explanation is that the activation of AMP-kinase in the liver by exercise increases fatty acid oxidation and decreases glucose production [29]. A previous animal study also suggested that daily exercise may decrease liver fat by reducing the activity of key enzymes of hepatic lipid synthesis, including acetyl coenzyme A carboxylase [30]. Enhanced fat oxidation leading to improvement in insulin sensitivity by physical activity is also considered a potential mechanism. Our results documenting the independent association between sufficient physical activity and NAFLD after adjusting for BMI also support this direct effect of exercise on the liver. Interestingly, the prevalence of DM was significantly higher in participants with NAFLD (20.7% vs. 5.3%, *p* < 0.001; Table 2) in the current study, suggesting that NAFLD and DM may share common pathophysiologic mechanisms, such as insulin resistance affected by physical activity.

Although physical activity is independently associated with NAFLD, obesity is still a well-established risk factor of NAFLD and can modulate the association between physical activity and NAFLD [7,12,13]. Specifically, obesity may be an important mediator of the association between NAFLD and physical activity in minimally active and inactive individuals. The current study also found that lean individuals had additional benefit from an intermediate level of physical activity. A small amount of physical activity could contribute to lower the risk of NAFLD in lean individuals, unlike in obese individuals. In addition, a higher BMI was significantly associated with the NAFLD, lean NAFLD, and abnormal levels of ALT and AST in the current study (Supplementary Tables S1–S4, respectively). Recent studies have shown the effectiveness of bariatric surgery to improve NAFLD by providing sustained long-term weight loss [31,32]. Therefore, losing weight by lifestyle change and/or bariatric surgery may be a synergistic therapy to improve the effects of physical activity on NAFLD [7].

As far as we know, there are few reports regarding the independent association of physical activity with the level of aminotransferases in patients with NAFLD. In this study, a higher physical activity level was associated with a lower level of ALT but not with AST regardless of using continuous (Figure 2) or dichotomous (Table 4) values of aminotransferases. This selective association of physical activity with ALT levels highlights the effect of physical activity on the liver because ALT is considered more sensitive and specific for the liver than AST [33]. In addition, ALT levels were significantly low only in HEPA active participants, which suggests that sufficient physical activity reaching the HEPA active category may be associated with an improvement in hepatocellular injury in individuals with NAFLD. A recent meta-analysis reported that physical activity, independent of diet change, was associated with a significant reduction in intrahepatic lipid content (standardized mean difference, −0.69) and with reductions in ALT (weighted mean difference, −3.30 IU/L) and AST (weighted mean difference, −4.85 IU/L) in patients with NAFLD [34]. Therefore, the underlying mechanism of the independent association between high physical activity and low ALT levels may be the reduction of intrahepatic fat by physical activity. Furthermore, improving blood glucose control and enhanced resistance to oxidant stress by physical activity may also contribute to the low ALT levels [35].

The optimal intensity and duration of physical activity required to be most beneficial to NAFLD remain to be determined. Since IPAQ-SF measures both the intensity and duration of aerobic activity, HEPA active participants are the most active individuals attaining at least 1500 MET-min/week of aerobic activity. Therefore, the results of our study indicate that the highest level of physical activity is required to lower the risk of NAFLD since the risk of NAFLD was not significantly decreased in the minimally active participants with intermediate levels of physical activity. However, in lean individuals, intermediate levels of physical activity can be beneficial to some degree to lower the risk of NAFLD. Furthermore, among the individuals with NAFLD, HEPA active participants also had the benefit of lower levels of ALT. Taken together, the results show that the physical activity level that meets the criteria of the HEPA active category could be considered as the optimal level of physical activity, which is associated with a low risk of NAFLD and nonalcoholic steatohepatitis, independent of obesity, diet, and comorbidities. The aerobic exercise programs meeting the HEPA active category can be prescribed among community-dwelling patients with NAFLD, emphasizing that an intermediate level of exercise program can be also beneficial in lean subjects with NAFLD. The results of our study are consistent with those of several other studies that have suggested the importance of high levels of physical activity in NAFLD [8,36,37].

The strength of the current study was the complex sampling design that can represent the general Korean population. Furthermore, we have considered various important confounding variables for NAFLD (e.g., BMI, central obesity, total calorie intake, lifestyle factors, and comorbidities) in addition to age and sex. However, our study has several limitations that should be acknowledged. First, the HSI was used as a surrogate for the diagnosis of NAFLD without an imaging test. However, the prevalence of NAFLD in our study (22.3%) is within the range of global prevalence (22–29% in the general population) [4] and is comparable to that of the previous study based on imaging-based diagnosis in the same country (28.2%) [10]. In addition, the HSI had been reliably employed in previous studies using KNHANES data [16]. Second, this study is a cross-sectional study that has the inherent limitations of not clarifying causal relationships. Future longitudinal cohort studies and randomized controlled trials are needed to elucidate the underlying causal relationships between physical activity and NAFLD. Third, although we included established confounders and used multiple progressively adjusted models, there may be unknown conditional associations between variables. We had to employ relatively old data since the necessary data for physical activity could not be obtained from recent surveys. In addition, because physical activity was assessed by questionnaires, misclassification or misreporting is possible. Finally, although the results of this study showed that physical activity is significantly associated with NAFLD, it is difficult to suggest what kind of physical activity is helpful and how long we should continue. Since IPAQ-SF assesses the aerobic activity, the role of resistance exercise could not be unveiled.

## 5. Conclusions

In conclusion, the level of physical activity is negatively associated with NAFLD and lean NAFLD independent of obesity and diet in Korean adults. Among the participants with NAFLD, the level of physical activity is also negatively associated with ALT levels independent of obesity and diet. The highest level of physical activity (HEPA active category) is required to benefit from these lower risks of NAFLD and ALT elevation. The lean adults have an additional benefit of lower risk of NAFLD from the intermediate level of physical activity.

## Figures and Tables

**Table 1:** Characteristics of participants by physical activity level (*n* = 32,391).

	HEPA Active (*n* = 8087)	Minimally Active (*n* = 11,112)	Inactive (*n* = 13,192)	*p* for Trend *
Age (years)	44.0 ± 0.3	44.9 ± 0.2	47.0 ± 0.2	<0.001
Male sex (%)	56.8 (0.7)	44.5 (0.6)	41.1 (0.6)	<0.001
BMI (kg/m^2^)	23.8 ± 0.1	23.4 ± 0.0	23.6 ± 0.0	<0.001
Central obesity (%)	21.6 (0.6)	21.9 (0.5)	24.4 (0.5)	<0.001
Total calorie intake (kcal/day)	2107.8 ± 15.1	1942.5 ± 11.7	1903.8 ± 10.4	<0.001
Sleep duration (h/day)	6.8 ± 0.0	6.8 ± 0.0	6.9 ± 0.0	0.002
Smoking (%)				<0.001
Never	52.1 (0.7)	60.2 (0.6)	60.2 (0.5)	
Past	22.3 (0.6)	19.8 (0.5)	18.6 (0.5)	
Current	25.6 (0.7)	20.0 (0.5)	21.3 (0.5)	
Alcohol (AUDIT score)	6.4 ± 0.1	5.3 ± 0.1	5.2 ± 0.1	<0.001
Education (years)				<0.001
≤6	15.1 (0.5)	17.7 (0.5)	22.9 (0.5)	
7–9	10.8 (0.4)	9.0 (0.3)	10.6 (0.4)	
10–12	44.1 (0.8)	37.2 (0.7)	35.2 (0.6)	
>12	30.0 (0.8)	36.1 (0.7)	31.3 (0.7)	
Diabetes mellitus (%)	7.4 (0.4)	8.8 (0.4)	9.6 (0.3)	<0.001
Cardiovascular disease (%)	2.6 (0.2)	3.5 (0.2)	3.7 (0.2)	<0.001
Hypertension (%)	24.4 (0.6)	24.5 (0.5)	27.0 (0.5)	<0.001
Arthritis (%)	8.9 (0.4)	10.1 (0.3)	12.1 (0.4)	<0.001
NAFLD (%)	21.6 (0.6)	21.7 (0.5)	23.4 (0.5)	0.016
Lean NAFLD (%)	2.7 (0.2)	3.9 (0.2)	4.1 (0.2)	<0.001
ALT (IU/L)	21.8 ± 0.3	21.0 ± 0.2	21.8 ± 0.2	0.022
AST (IU/L)	22.2 ± 0.2	21.1 ± 0.1	21.6 ± 0.1	<0.001

Values are the weighted means ± SE or weighted percentage (SE), as appropriate. * *p* values by analysis of variance for continuous variables and the Chi square test for categorical variables. HEPA, health-enhancing physical activity; BMI, body mass index; AUDIT, Alcohol Use Disorders Identification Test; NAFLD, non-alcoholic fatty liver disease; ALT: alanine aminotransferase, AST: aspartate aminotransferase.

**Table 2:** Characteristics of participants by the presence of NAFLD (*n* = 32,391).

	No NAFLD (*n* = 25,423)	NAFLD (*n* = 6968)	*p* *
Age (years)	45.4 ± 0.2	45.9 ± 0.2	0.032
Male sex (%)	44.5 (0.4)	52.9 (0.8)	<0.001
BMI (kg/m^2^)	22.4 ± 0.0	27.7 ± 0.1	<0.001
Central obesity (%)	11.0 (0.3)	63.9 (0.8)	<0.001
Total calorie intake (kcal/day)	1961.6 ± 8.2	2001.7 ± 16.0	0.018
Sleep duration (h/day)	6.9 ± 0.0	6.8 ± 0.0	0.001
Smoking (%)			<0.001
Non	59.4 (0.4)	53.2 (0.8)	
Ex	19.9 (0.3)	20.3 (0.6)	
Current	20.7 (0.4)	26.6 (0.7)	
Alcohol (AUDIT score)	5.5 ± 0.0	5.7 ± 0.1	0.113
Education (years)			<0.001
≤6	18.5 (0.4)	21.2 (0.6)	
7–9	9.9 (0.3)	11.0 (0.5)	
10–12	38.4 (0.5)	37.4 (0.8)	
>12	33.3 (0.5)	30.4 (0.9)	
Diabetes mellitus (%)	5.3 (0.2)	20.7 (0.6)	<0.001
Cardiovascular disease (%)	3.1 (0.1)	4.1 (0.1)	<0.001
Hypertension (%)	21.7 (0.4)	38.4 (0.8)	<0.001
Arthritis (%)	9.7 (0.2)	13.6 (0.5)	<0.001
ALT (IU/L)	17.2 ± 0.1	36.5 ± 0.5	<0.001
AST (IU/L)	20.5 ± 0.1	25.5 ± 0.2	<0.001
Physical activity level (%)			0.016
HEPA active	26.5 (0.4)	25.3 (0.7)	
Minimally active	34.5 (0.4)	33.2 (0.7)	
Inactive	39.0 (0.5)	41.5 (0.8)	

Values are the weighted means ± SE or weighted percentage (SE), as appropriate. * *p* values by Student’s *t*-tests for continuous variables and the Chi square test for categorical variables. NAFLD, non-alcoholic fatty liver disease; BMI, body mass index; AUDIT, Alcohol Use Disorders Identification Test; ALT, alanine aminotransferase; AST, aspartate aminotransferase; HEPA, health-enhancing physical activity; MET, metabolic equivalent.

**Table 3:** Adjusted OR for the independent association of physical activity with NAFLD.

	Inactive	Minimally Active	HEPA Active	*p* for Trend *
NAFLD (vs. No NAFLD, *n* = 32,391)				
Model 1	1.0	0.9 (0.8–0.97)	0.9 (0.8–0.9)	0.001
Model 2	1.0	0.9 (0.8–1.03)	0.7 (0.6–0.7)	<0.001
Model 3	1.0	0.9 (0.8–1.04)	0.7 (0.6–0.8)	<0.001
Model 4	1.0	0.9 (0.8–1.03)	0.7 (0.6–0.8)	<0.001
Model 5	1.0	0.9 (0.8–1.02)	0.7 (0.6–0.8)	<0.001
Lean NAFLD (vs. Lean control subjects without NAFLD, *n* = 22,312)				
Model 1	1.0	0.9 (0.8–1.1)	0.6 (0.5–0.7)	<0.001
Model 2	1.0	0.9 (0.7–1.1)	0.5 (0.4–0.6)	<0.001
Model 3	1.0	0.8 (0.7–0.98)	0.5 (0.4–0.6)	<0.001
Model 4	1.0	0.8 (0.6–0.97)	0.5 (0.4–0.7)	<0.001
Model 5	1.0	0.8 (0.6–0.98)	0.5 (0.4–0.7)	<0.001

Values are the OR (95% CI). * *p* values by complex-samples logistic regression model. Model 1, adjusted for age and sex. Model 2, adjusted for model 1 plus BMI. Model 3, adjusted for model 2 plus total calorie intake, sleep duration, smoking, alcohol, education level. Model 4, adjusted for model 3 plus diabetes mellitus, cardiovascular disease, hypertension, arthritis. Model 5, adjusted for model 4 plus central obesity. OR, odds ratio; NAFLD, non-alcoholic fatty liver disease; HEPA, health-enhancing physical activity; BMI, body mass index.

**Table 4:** Adjusted OR for the independent association of physical activity with abnormal levels * of ALT and AST among participants with NAFLD (*n* = 6968).

	Inactive	Minimally Active	HEPA Active	*p* for Trend ^†^
Abnormal ALT				
Model 1	1.0	1.0 (0.8–1.1)	0.8 (0.7–0.9)	0.013
Model 2	1.0	1.0 (0.8–1.1)	0.8 (0.7–0.9)	0.020
Model 3	1.0	1.0 (0.8–1.1)	0.8 (0.6–0.9)	0.007
Model 4	1.0	1.0 (0.8–1.2)	0.8 (0.6–0.9)	0.007
Model 5	1.0	1.0 (0.8–1.1)	0.8 (0.6–0.9)	0.006
Abnormal AST				
Model 1	1.0	0.9 (0.8–1.1)	0.9 (0.8–1.1)	0.621
Model 2	1.0	0.9 (0.8–1.1)	0.9 (0.8–1.1)	0.652
Model 3	1.0	0.9 (0.8–1.1)	0.9 (0.7–1.1)	0.409
Model 4	1.0	0.9 (0.8–1.1)	0.9 (0.7–1.1)	0.451
Model 5	1.0	0.9 (0.8–1.1)	0.9 (0.7–1.1)	0.427

Values are the OR (95% CI). * Defined as >34 IU/L in men or >24 IU/L in women for ALT and >32 IU/L in men or >26 IU/L in women for AST. ^†^ *p* values by complex-samples logistic regression model. Model 1 adjusted for age and sex. Model 2, adjusted for model 1 plus BMI. Model 3, adjusted for model 2 plus total calorie intake, sleep duration, smoking, alcohol, education level. Model 4, adjusted for model 3 plus diabetes mellitus, cardiovascular disease, hypertension, arthritis. Model 5, adjusted for model 4 plus central obesity. OR, odds ratio; ALT, alanine aminotransferase; AST, aspartate aminotransferase; NAFLD, non-alcoholic fatty liver disease; HEPA, health-enhancing physical activity; BMI, body mass index.

## Supplementary Materials

The following are available online at https://www.mdpi.com/2077-0383/8/7/1013/s1, Table S1: Sociodemographic, lifestyle, and clinical correlates for NAFLD, Table S2: Sociodemographic, lifestyle, and clinical correlates for lean NAFLD, Table S3: Sociodemographic, lifestyle, and clinical correlates for abnormal levels of ALT among participants with NAFLD, Table S4: Sociodemographic, lifestyle, and clinical correlates for abnormal levels of AST among participants with NAFLD.

## References

[1] Chalasani N., Younossi Z., Lavine J.E., Charlton M., Cusi K., Rinella M., Harrison S.A., Brunt E.M., Sanyal A.J. (2018). The diagnosis and management of nonalcoholic fatty liver disease: Practice guidance from the american association for the study of liver diseases. Hepatology.

[2] Duseja A., Chalasani N. (2013). Epidemiology and risk factors of nonalcoholic fatty liver disease (NAFLD). Hepatol. Int..

[3] Younossi Z., Anstee Q.M., Marietti M., Hardy T., Henry L., Eslam M., George J., Bugianesi E. (2018). Global burden of nafld and nash: Trends, predictions, risk factors and prevention. Nat. Rev. Gastroenterol. Hepatol..

[4] Younossi Z.M., Koenig A.B., Abdelatif D., Fazel Y., Henry L., Wymer M. (2016). Global epidemiology of nonalcoholic fatty liver disease-meta-analytic assessment of prevalence, incidence, and outcomes. Hepatology.

[5] Denkmayr L., Feldman A., Stechemesser L., Eder S.K., Zandanell S., Schranz M., Strasser M., Huber-Schonauer U., Buch S., Hampe J. (2018). Lean patients with non-alcoholic fatty liver disease have a severe histological phenotype similar to obese patients. J. Clin. Med..

[6] Golabi P., Paik J., Fukui N., Locklear C.T., de Avilla L., Younossi Z.M. (2019). Patients with lean nonalcoholic fatty liver disease are metabolically abnormal and have a higher risk for mortality. Clin. Diabetes.

[7] European Association for the Study of the Liver (EASL), European Association for the Study of Diabetes (EASD), European Association for the Study of Obesity (EASO) (2016). EASL-EASD-EASO clinical practice guidelines for the management of non-alcoholic fatty liver disease. J. Hepatol..

[8] Qiu S., Cai X., Sun Z., Li L., Zugel M., Steinacker J.M., Schumann U. (2017). Association between physical activity and risk of nonalcoholic fatty liver disease: A meta-analysis. Therap. Adv. Gastroenterol..

[9] Kwak M.S., Kim D., Chung G.E., Kim W., Kim Y.J., Yoon J.H. (2015). Role of physical activity in nonalcoholic fatty liver disease in terms of visceral obesity and insulin resistance. Liver Int..

[10] Ryu S., Chang Y., Jung H.S., Yun K.E., Kwon M.J., Choi Y., Kim C.W., Cho J., Suh B.S., Cho Y.K. (2015). Relationship of sitting time and physical activity with non-alcoholic fatty liver disease. J. Hepatol..

[11] Gerber L., Otgonsuren M., Mishra A., Escheik C., Birerdinc A., Stepanova M., Younossi Z.M. (2012). Non-alcoholic fatty liver disease (nafld) is associated with low level of physical activity: A population-based study. Aliment. Pharmacol. Ther..

[12] Zelber-Sagi S., Nitzan-Kaluski D., Goldsmith R., Webb M., Zvibel I., Goldiner I., Blendis L., Halpern Z., Oren R. (2008). Role of leisure-time physical activity in nonalcoholic fatty liver disease: A population-based study. Hepatology.

[13] Long M.T., Pedley A., Massaro J.M., Hoffmann U., Esliger D.W., Vasan R.S., Fox C.S., Murabito J.M. (2015). Hepatic steatosis is associated with lower levels of physical activity measured via accelerometry. Obes. Silver Spring.

[14] Korean Centers for Disease Control and Prevention Korea National Health and Nutrition Examination Surveys. http://knhanes.cdc.go.kr.

[15] Lee J.H., Kim D., Kim H.J., Lee C.H., Yang J.I., Kim W., Kim Y.J., Yoon J.H., Cho S.H., Sung M.W. (2010). Hepatic steatosis index: A simple screening tool reflecting nonalcoholic fatty liver disease. Dig. Liver Dis..

[16] Lee Y.H., Jung K.S., Kim S.U., Yoon H.J., Yun Y.J., Lee B.W., Kang E.S., Han K.H., Lee H.C., Cha B.S. (2015). Sarcopaenia is associated with nafld independently of obesity and insulin resistance: Nationwide surveys (knhanes 2008–2011). J. Hepatol..

[17] Oh J.Y., Yang Y.J., Kim B.S., Kang J.H. (2007). Validity and reliability of korean version of international physical activity questionnaire (ipaq) short form. Korean J. Fam. Med..

[18] Fogelholm M., Malmberg J., Suni J., Santtila M., Kyrolainen H., Mantysaari M., Oja P. (2006). International physical activity questionnaire: Validity against fitness. Med. Sci. Sports Exerc..

[19] Fan J.G., Kim S.U., Wong V.W. (2017). New trends on obesity and nafld in asia. J. Hepatol..

[20] Lee S.Y., Park H.S., Kim D.J., Han J.H., Kim S.M., Cho G.J., Kim D.Y., Kwon H.S., Kim S.R., Lee C.B. (2007). Appropriate waist circumference cutoff points for central obesity in korean adults. Diabetes Res. Clin. Pract..

[21] Sohn W., Jun D.W., Kwak M.J., Park Q., Lee K.N., Lee H.L., Lee O.Y., Yoon B.C., Choi H.S. (2013). Upper limit of normal serum alanine and aspartate aminotransferase levels in korea. J. Gastroenterol. Hepatol..

[22] Koehler E.M., Schouten J.N., Hansen B.E., van Rooij F.J., Hofman A., Stricker B.H., Janssen H.L. (2012). Prevalence and risk factors of non-alcoholic fatty liver disease in the elderly: Results from the rotterdam study. J. Hepatol..

[23] Liu P., Xu Y., Tang Y., Du M., Yu X., Sun J., Xiao L., He M., Wei S., Yuan J. (2017). Independent and joint effects of moderate alcohol consumption and smoking on the risks of non-alcoholic fatty liver disease in elderly chinese men. PLoS ONE.

[24] Peng K., Lin L., Wang Z., Ding L., Huang Y., Wang P., Xu Y., Lu J., Xu M., Bi Y. (2017). Short sleep duration and longer daytime napping are associated with non-alcoholic fatty liver disease in chinese adults. J. Diabetes.

[25] Targher G., Day C.P., Bonora E. (2010). Risk of cardiovascular disease in patients with nonalcoholic fatty liver disease. N. Engl. J. Med..

[26] Habib G.S., Saliba W.R. (2001). Arthritis associated with non-alcoholic steatohepatitis. Ann. Rheum. Dis..

[27] Thoma C., Day C.P., Trenell M.I. (2012). Lifestyle interventions for the treatment of non-alcoholic fatty liver disease in adults: A systematic review. J. Hepatol..

[28] Holt H.B., Wild S.H., Wareham N., Ekelund U., Umpleby M., Shojaee-Moradie F., Holt R.I., Phillips D.I., Byrne C.D. (2007). Differential effects of fatness, fitness and physical activity energy expenditure on whole-body, liver and fat insulin sensitivity. Diabetologia.

[29] Kemp B.E., Mitchelhill K.I., Stapleton D., Michell B.J., Chen Z.P., Witters L.A. (1999). Dealing with energy demand: The amp-activated protein kinase. Trends Biochem. Sci..

[30] Rector R.S., Thyfault J.P., Morris R.T., Laye M.J., Borengasser S.J., Booth F.W., Ibdah J.A. (2008). Daily exercise increases hepatic fatty acid oxidation and prevents steatosis in otsuka long-evans tokushima fatty rats. Am. J. Physiol. Gastrointest. Liver Physiol..

[31] Nickel F., Tapking C., Benner L., Sollors J., Billeter A.T., Kenngott H.G., Bokhary L., Schmid M., von Frankenberg M., Fischer L. (2018). Bariatric surgery as an efficient treatment for non-alcoholic fatty liver disease in a prospective study with 1-year follow-up: Bariscan study. Obes. Surg..

[32] Von Schonfels W., Beckmann J.H., Ahrens M., Hendricks A., Rocken C., Szymczak S., Hampe J., Schafmayer C. (2018). Histologic improvement of nafld in patients with obesity after bariatric surgery based on standardized nas (nafld activity score). Surg. Obes. Relat. Dis..

[33] Pratt D.S., Kaplan M.M. (2000). Evaluation of abnormal liver-enzyme results in asymptomatic patients. N. Engl. J. Med..

[34] Orci L.A., Gariani K., Oldani G., Delaune V., Morel P., Toso C. (2016). Exercise-based interventions for nonalcoholic fatty liver disease: A meta-analysis and meta-regression. Clin. Gastroenterol. Hepatol..

[35] Shephard R.J., Johnson N. (2015). Effects of physical activity upon the liver. Eur. J. Appl. Physiol..

[36] Tsunoda K., Kai Y., Kitano N., Uchida K., Kuchiki T., Nagamatsu T. (2016). Impact of physical activity on nonalcoholic steatohepatitis in people with nonalcoholic simple fatty liver: A prospective cohort study. Prev. Med..

[37] Kistler K.D., Brunt E.M., Clark J.M., Diehl A.M., Sallis J.F., Schwimmer J.B., Group N.C.R. (2011). Physical activity recommendations, exercise intensity, and histological severity of nonalcoholic fatty liver disease. Am. J. Gastroenterol..

