# Supplementary material for: Independent Association of Physical Activity with Nonalcoholic Fatty Liver Disease and Alanine Aminotransferase Levels
Source: J Clin Med. 2019 Jul 10;8(7):1013. doi: 10.3390/jcm8071013 (PMC6678247; doi:10.3390/jcm8071013)
Supplement: Supplementary file 1 [file jcm-08-01013-s001.pdf]

Supplementary Table 1. Sociodemographic, lifestyle, and clinical correlates for NAFLD (n = 32,391).

	Model 1	Model 2	Model 3	Model 4	Model 5
Age (per 10 years)	1.03 (1.01–1.05)	0.9 (0.8–0.9)	0.8 (0.8–0.9)	0.7 (0.7–0.8)	0.7 (0.7–0.7)
Male sex	1.4 (1.3–1.5)	1.1 (1.01–1.2)	1.2 (0.99–1.4)	1.1 (0.9–1.3)	1.1 (0.9–1.3)
BMI (per 1 kg/m^2)		2.4 (2.3–2.4)	2.4 (2.3–2.5)	2.5 (2.4–2.5)	2.4 (2.3–2.5)
Central obesity					1.4 (1.2–1.6)
Total calorie intake (per 100 kcal/day)			1.0 (0.99–1.003)	1.0 (0.99–1.01)	1.0 (0.99–1.01)
Sleep duration (per 1 h/day)			1.0 (0.99–1.1)	1.0 (0.97–1.1)	1.0 (0.97–1.1)
Smoking					
Never			1.0	1.0	1.0
Past			1.1 (0.9–1.3)	1.1 (0.9–1.3)	1.0 (0.9–1.2)
Current			1.5 (1.3–1.8)	1.5 (1.2–1.8)	1.5 (1.2–1.8)
Alcohol (per 1 AUDIT score)			1.0 (0.96–0.98)	1.0 (0.96–0.98)	1.0 (0.95–0.98)
Education					
≤6			1.0	1.0	1.0
7–9			1.0 (0.8–1.2)	1.0 (0.8–1.2)	1.0 (0.8–1.2)
10–12			0.9 (0.8–1.1)	1.0 (0.8–1.2)	1.0 (0.8–1.2)
>12			1.0 (0.8–1.2)	1.1 (0.9–1.3)	1.1 (0.9–1.3)
Diabetes mellitus				8.2 (6.9–9.8)	8.2 (6.9–9.8)
Cardiovascular disease				0.9 (0.7–1.1)	0.9 (0.7–1.1)
Hypertension				1.1 (0.9–1.2)	1.1 (0.9–1.2)
Arthritis				1.0 (0.9–1.2)	1.0 (0.9–1.2)

Values are the OR (95% CI). Model 1, adjusted for physical activity, age and sex. Model 2, adjusted for model 1 plus BMI. Model 3, adjusted for model 2 plus total calorie intake, sleep duration, smoking, alcohol, education level. Model 4, adjusted for model 3 plus diabetes mellitus, cardiovascular disease, hypertension, arthritis. Model 5, adjusted for model 4 plus central obesity. NAFLD, non-alcoholic fatty liver disease; OR, odds ratio; BMI, body mass index; AUDIT, Alcohol Use Disorders Identification Test.

Supplementary Table 2. Sociodemographic, lifestyle, and clinical correlates for lean NAFLD (n = 22,312).

	Model 1	Model 2	Model 3	Model 4	Model 5
Age (per 10 years)	1.0 (0.98–1.1)	0.9 (0.9–0.9)	0.8 (0.8–0.9)	0.8 (0.7–0.8)	0.8 (0.7–0.8)
Male sex	2.0 (1.7–2.4)	1.5 (1.3–1.8)	1.8 (1.4–2.3)	1.6 (1.2–2.0)	1.6 (1.2–2.0)
BMI (per 1 kg/m^2)		2.2 (2.0–2.4)	2.2 (2.0–2.3)	2.2 (2.0–2.4)	2.2 (2.0–2.4)
Central obesity					1.3 (0.97–1.7)
Total calorie intake (per 100 kcal/day)			1.0 (0.98–1.003)	1.0 (0.99–1.01)	1.0 (0.98–1.01)
Sleep duration (per 1 h/day)			1.0 (0.98–1.1)	1.0 (0.97–1.1)	1.0 (0.97–1.1)
Smoking					
Never			1.0	1.0	1.0
Past			1.2 (0.9–1.6)	1.2 (0.9–1.6)	1.2 (0.9–1.6)
Current			1.3 (0.97–1.7)	1.3 (0.96–1.7)	1.3 (0.96–1.7)
Alcohol (per 1 AUDIT score)			1.0 (0.9–0.98)	1.0 (0.9–0.98)	1.0 (0.9–0.98)
Education					
≤6			1.0	1.0	1.0
7–9			0.9 (0.7–1.3)	0.9 (0.6–1.3)	0.9 (0.6–1.3)
10–12			1.1 (0.8–1.5)	1.1 (0.8–1.5)	1.1 (0.8–1.5)
>12			1.1 (0.8–1.5)	1.2 (0.8–1.6)	1.2 (0.9–1.7)
Diabetes mellitus				7.2 (5.6–9.2)	7.2 (5.6–9.2)
Cardiovascular disease				0.8 (0.5–1.2)	0.8 (0.5–1.2)
Hypertension				1.2 (0.9–1.5)	1.2 (0.9–1.5)
Arthritis				0.8 (0.6–1.1)	0.8 (0.6–1.1)

Values are the OR (95% CI). Model 1, adjusted for physical activity, age and sex. Model 2, adjusted for model 1 plus BMI. Model 3, adjusted for model 2 plus total calorie intake, sleep duration, smoking, alcohol, education level. Model 4, adjusted for model 3 plus diabetes mellitus, cardiovascular disease, hypertension, arthritis. Model 5, adjusted for model 4 plus central obesity. NAFLD, non-alcoholic fatty liver disease; OR, odds ratio; BMI, body mass index; AUDIT, Alcohol Use Disorders Identification Test.

Supplementary Table 3. Sociodemographic, lifestyle, and clinical correlates for abnormal levels of ALT* among participants with NAFLD (*n* = 6968).

	Model 1	Model 2	Model 3	Model 4	Model 5
Age (per 10 years)	1.0 (0.9–1.01)	1.0 (0.9–0.9996)	0.9 (0.9–1.01)	0.9 (0.8–0.98)	0.9 (0.8–0.98)
Male sex	1.4 (1.3–1.6)	1.4 (1.2–1.6)	1.5 (1.2–1.8)	1.4 (1.1–1.7)	1.4 (1.2–1.8)
BMI (per 1 kg/m^2)		0.9 (0.9–0.97)	0.9 (0.9–0.97)	0.9 (0.9–0.97)	0.9 (0.9–0.97)
Central obesity					1.1 (0.9–1.3)
Total calorie intake (per 100 kcal/day)			1.0 (0.99–1.004)	1.0 (0.99–1.004)	1.0 (0.99–1.004)
Sleep duration (per 1 h/day)			1.0 (0.96–1.1)	1.0 (0.95–1.1)	1.0 (0.95–1.1)
Smoking					
Never			1.0	1.0	1.0
Past			0.9 (0.7–1.1)	0.9 (0.7–1.2)	0.9 (0.7–1.2)
Current			1.0 (0.8–1.2)	1.0 (0.8–1.2)	1.0 (0.8–1.2)
Alcohol (per 1 AUDIT score)			1.0 (0.99–1.02)	1.0 (0.99–1.02)	1.0 (0.99–1.02)
Education					
≤6			1.0	1.0	1.0
7–9			0.9 (0.7–1.2)	0.9 (0.7–1.2)	0.9 (0.7–1.2)
10–12			1.0 (0.7–1.2)	1.0 (0.8–1.2)	1.0 (0.8–1.2)
>12			1.0 (0.8–1.3)	1.0 (0.8–1.3)	1.0 (0.8–1.3)
Diabetes mellitus				1.1 (0.9–1.4)	1.1 (0.9–1.4)
Cardiovascular disease				0.8 (0.6–1.1)	0.8 (0.6–1.1)
Hypertension				1.2 (1.1–1.5)	1.2 (1.1–1.5)
Arthritis				1.0 (0.8–1.2)	1.0 (0.8–1.2)

Values are the OR (95% CI). *Defined as >34 IU/L in men or >24 IU/L in women. Model 1, adjusted for physical activity, age and sex. Model 2, adjusted for model 1 plus BMI. Model 3, adjusted for model 2 plus total calorie intake, sleep duration, smoking, alcohol, education level. Model 4, adjusted for model 3 plus diabetes mellitus, cardiovascular disease, hypertension, arthritis. Model 5, adjusted for model 4 plus central obesity. ALT, alanine aminotransferase; NAFLD, non-alcoholic fatty liver disease; OR, odds ratio; BMI, body mass index; AUDIT, Alcohol Use Disorders Identification Test.

Supplementary Table 4. Sociodemographic, lifestyle, and clinical correlates for abnormal levels of AST* among participants with NAFLD (*n* = 6968).

	Model 1	Model 2	Model 3	Model 4	Model 5
Age (per 10 years)	1.1 (1.01–1.1)	1.1 (1.03–1.1)	1.1 (0.99–1.2)	1.0 (0.9–1.1)	1.0 (0.9–1.1)
Male sex	0.9 (0.8–1.04)	0.9 (0.8–1.1)	1.0 (0.8–1.3)	0.9 (0.7–1.2)	0.9 (0.7–1.2)
BMI (per 1 kg/m^2)		1.1 (1.03–1.1)	1.1 (1.03–1.1)	1.1 (1.02–1.1)	1.1 (1.02–1.1)
Central obesity					1.0 (0.8–1.2)
Total calorie intake (per 100 kcal/day)			1.0 (0.99–1.01)	1.0 (0.99–1.01)	1.0 (0.99–1.01)
Sleep duration (per 1 h/day)			1.0 (0.9–1.1)	1.0 (0.9–1.1)	1.0 (0.9–1.1)
Smoking					
Never			1.0	1.0	1.0
Past			1.0 (0.7–1.2)	1.0 (0.7–1.2)	1.0 (0.7–1.2)
Current			0.9 (0.7–1.1)	0.9 (0.7–1.1)	0.9 (0.7–1.1)
Alcohol (per 1 AUDIT score)			1.0 (0.997–1.03)	1.0 (0.996–1.03)	1.0 (0.996–1.03)
Education					
≤6			1.0	1.0	1.0
7–9			1.0 (0.8–1.4)	1.0 (0.8–1.4)	1.0 (0.8–1.4)
10–12			1.0 (0.7–1.3)	1.0 (0.8–1.3)	1.0 (0.8–1.3)
>12			0.9 (0.7–1.2)	1.0 (0.7–1.3)	1.0 (0.7–1.3)
Diabetes mellitus				1.5 (1.2–1.8)	1.5 (1.2–1.8)
Cardiovascular disease				1.2 (0.8–1.7)	1.2 (0.8–1.7)
Hypertension				1.1 (0.9–1.4)	1.1 (0.9–1.4)
Arthritis				1.0 (0.8–1.2)	1.0 (0.8–1.2)

Values are the OR (95% CI). *Defined as >32 IU/L in men or >26 IU/L in women. Model 1, adjusted for physical activity, age and sex. Model 2, adjusted for model 1 plus BMI. Model 3, adjusted for model 2 plus total calorie intake, sleep duration, smoking, alcohol, education level. Model 4, adjusted for model 3 plus diabetes mellitus, cardiovascular disease, hypertension, arthritis. Model 5, adjusted for model 4 plus central obesity. ALT, alanine aminotransferase; NAFLD, non-alcoholic fatty liver disease; OR, odds ratio; BMI, body mass index; AUDIT, Alcohol Use Disorders Identification Test.
